# FINANCIAL SCAMS TARGETING OLDER ADULTS DURING COVID-19

**DOI:** 10.1093/geroni/igac059.1181

**Published:** 2022-12-20

**Authors:** Scott Beach, Sara Czaja, Richard Schulz, David Loewenstein, Peter Lichtenberg

**Affiliations:** University Of Pittsburgh, Pittsburgh, Pennsylvania, United States; Weill Cornell Medicine, New York City, New York, United States; University of Pittsburgh, Pittsburgh, Pennsylvania, United States; University of Miami, Miami, Florida, United States; Wayne State University, Detroit, Michigan, United States

## Abstract

The COVID pandemic afforded financial scammers with new opportunities to target older adults. This paper presents data from a telephone survey conducted June–September, 2020 with 380 participants from a larger National Institute of Aging study examining financial exploitation among older adults. The survey assessed COVID-related scams in three areas: (1) products, testing, treatments; (2) financial assistance (e.g., stimulus checks); and (3) charities. Questions focused on scam exposure / attempts, mode of contact, responses, and whether the older adult reported it to someone. The sample (284 Pittsburgh; 96 New York City) was 64% female; mean age = 73.6; and 47% White, 41% African American, 12% Hispanic. Across all scam types, 18.4% reported scam attempts / exposure; 24% of those exposed engaged / responded without getting scammed (11%); or were actually scammed (13%); and 40% told someone about it. The most frequent modes of contact were: telephone (54%), internet / email (40%), or mail (29%). Controlling for socio-demographics, participants from NYC were more likely to be exposed (OR = 1.91; p=.036); as were those reporting more loneliness (8-item UCLA scale; OR = 1.06; p=.042); and those reporting that COVID had worsened their emotional well-being to a greater extent (OR = 1.57; p=.032). Older adults who were more socially isolated / lonely were also more likely to have been scammed and less like to tell anyone about it. Psychosocial factors play an important role in exposure and response to scams during pandemics. Implications for policy, intervention, and general scam susceptibility are discussed.

